# Neuroprotective properties of dehydroepiandrosterone-sulfate and its relationship to interleukin 6 after aneurysmal subarachnoid hemorrhage: a prospective cohort study

**DOI:** 10.1186/s13054-015-0954-1

**Published:** 2015-05-21

**Authors:** Anke Höllig, Miriam Thiel, Birgit Stoffel-Wagner, Mark Coburn, Hans Clusmann

**Affiliations:** Department of Neurosurgery, RWTH Aachen University, Pauwelsstr. 30, D-52074 Aachen, Germany; Department of Anesthesiology, RWTH Aachen University, Aachen, Germany; Department of Pediatrics, GFO Kliniken Bonn Zweigstelle St. Marien-Hospital, Bonn, Germany; Institute of Clinical Chemistry and Clinical Pharmacology, University of Bonn, Bonn, Germany

## Abstract

**Introduction:**

The established neuroprotective property of the sex steroid precursor dehydroepiandrosterone-sulfate (DHEAS) has not yet been investigated in the context of aneurysmal subarachnoid hemorrhage (aSAH). The influence of DHEAS on inflammatory response resulting in modulation of interleukin 6 (IL-6) synthesis has been shown. Here, we evaluate DHEAS serum levels after aSAH (day 0–14) and levels of IL-6 related to functional outcome at discharge and at six months.

**Methods:**

A complete data set (DHEAS and IL-6 serum levels for days 0, 1, 4, 7, 10 and 14 after aSAH) and outcome assessment at discharge according to modified Rankin Scale score (mRS) was available for 53 patients of the initially screened cohort (*n* = 109). Outcome assessment six months after aSAH was obtained from 41 patients. Logarithmized levels of DHEAS and IL-6 were related to dichotomized functional outcome either assessed at discharge or at six months. A mixed between-within subjects ANOVA was applied for statistical analysis (SPSS 21.0).

**Results:**

DHEAS and IL-6 levels across time were related to functional outcome. Regarding outcome assessment at discharge and at six months after aSAH, DHEAS levels (transformed to square root for statistical purposes) were considerably higher in patients with favorable outcome (mRS 0–2) (*p* = .001; *p* = .020). Inversely, in patients with favorable outcome either at discharge or six months after aSAH, lower IL-6 levels (logarithmized for statistical purposes) were observed across time (both *p* < .001).

**Conclusion:**

We provide new evidence that DHEAS is associated with protective properties resulting in improvement of functional outcome after aSAH, possibly by influencing the inflammatory response after aSAH shown in the decreasing IL-6 serum levels. But the results for outcome six months after SAH are limited due to a high drop-out rate.

**Electronic supplementary material:**

The online version of this article (doi:10.1186/s13054-015-0954-1) contains supplementary material, which is available to authorized users.

## Introduction

Aneurysmal subarachnoid hemorrhage (aSAH) is a severe condition. Despite a gradual decrease over the last decades, case fatality still accounts for 40 % [1]. Patients affected are younger (mean 52 years) than those struck by ischemic stroke. Therefore, and due to a high rate of patients with permanent deficits, individual implications as well as the costs for society are enormous [2, 3]. There is evidence that hormonal influence, specifically neuroprotective effects achieved by sex steroids, may alleviate the brain injury generated by aSAH [4–6]. While the clinical finding that female sex is a risk factor for aSAH consolidated this hypothesis, it has only been demonstrated in older cohorts (>50 years) [7–9]. However, the data are still conflicting with no definite consensus on the possible influence and mechanism of sex steroid action after aSAH [10].

Dehydroepiandrosterone sulfate (DHEAS) is the sulfate ester of dehydroepiandrosterone (DHEA); both are multifunctional steroid hormones and precursors of sex steroids constituting the most abundant human steroid hormones [11]. DHEAS easily crosses the blood brain barrier [12]. Within the central nervous system, it displays various effects contributing to neuroprotection, such as anti-inflammatory, pro-survival and anti-glucocorticoid properties [11]. Experimental and clinical studies have repeatedly reported the neuroprotective properties of DHEAS [13–16].

DHEAS also influences neuroinflammation: synthesis of IL-6, a major cytokine involved in neuroinflammation [17], may be inhibited by DHEAS [18] and a negative correlation of DHEAS with IL-6 has been shown in vitro [19]. In a physiological state, IL-6 plays a role in neuropoiesis and neurogenesis [20, 21], whereas under pathophysiological conditions, it is one of the major stimuli of neuroinflammatory response [17, 22–24]. With respect to aSAH, there is evidence that higher early serum IL-6 levels predict an unfavorable outcome most probably by contributing to the early inflammatory response after aSAH as part of early brain injury [25].

Given the neuroprotective effects of DHEAS and its interaction with IL-6, this linkage of the neuroendocrine with the neuroinflammatory system may exert neuroprotective properties via DHEAS-mediated attenuation of the early inflammatory response. Thus, we aimed to assess the role of DHEAS and its relation to IL-6 after aSAH. By analyzing DHEAS and IL-6 serum levels across time and relating these results to functional outcome (determined by modified Rankin scale score (mRs) at discharge and 6 months after aSAH), we aimed to elucidate the potential beneficial effects of DHEAS and its relationship with neuroinflammation.

## Methods

The prospective study was approved by the local ethics committee (Ethikkommission an der Medizinischen Fakultät der Rheinischen Friedrich-Wilhelms-Universität Bonn, Germany; EK 199/08). Written informed consent was obtained from patients or legal representatives. Within a 21-month period (February 2009 to November 2010) 109 consecutive patients with a proven aSAH were screened for eligibility. Patients were not eligible if they were younger than 18 years, enrolled in other clinical trials, admitted more than 12 h after onset or if informed consent could not be obtained. In total, 81 patients (74 %) were included. At admission, demographic data, severity score for aSAH according to the World Federation of Neurosurgical Societies (WFNS) classification and time span to clinical ictus were assessed. Treatment of patients was carried out according to standardized guidelines [26]. Serum levels of DHEAS (lower limit of detection (LOD) 150 ng/ml) and IL-6 (LOD 2 pg/ml) were measured directly at admission and on days 1, 4, 7, 10 and 14 after aSAH (always in the morning) in line with routine diagnostics at the Department of Clinical Chemistry and Clinical Pharmacology, University of Bonn. Samples were centrifuged for 10 minutes at 2000 g prior to processing.

At discharge and 6 months thereafter, functional outcome according to the mRS was determined. For statistical analysis, the mRS was dichotomized (mRS 0–2 = favorable, mRS 3–6 = unfavorable). Scores according to the WFNS grading scale were also dichotomized with WFNS 1–3 defined as mild hemorrhage and WFNS 4 and 5 defined as severe hemorrhage. Furthermore, patient age was dichotomized to assess age-dependent differences (age <55 years vs >55 years).

Normality of the distribution was tested by the Kolmogorov–Smirnov test. Data were checked for outliers, and the data for one patient had to be removed (100-fold increase of IL-6 levels at day 1 compared to other results from the same patient). For descriptive analysis, mean, minimum and maximum values, standard deviation (SD) and 95 % confidence interval (95 % CI) were calculated. Graphs show mean values and 95 % CI. For skewed data, the mixed between-within subjects analysis of variance (ANOVA) data were tested for normal distribution. Sphericity assumption was checked; violation (significant Mauchly test) was compensated for using Greenhouse-Geisser or Huyn-Feldt corrected degrees of freedom as alternative requirements for appropriate analysis. Data were transformed (using logarithm (log) or square root (sqrt)) to ensure homogeneity of variances and sphericity (or its respective alternative requirements). Subgroup analyses (for gender, age and WFNS score) were only performed related to outcome at discharge, due to the small sample size. A *p*-value less than 0.05 was regarded to be statistically significant. Statistical analysis was performed using IBM SPSS 21.0 software. All graphs were drawn using GraphPad Prism 6.

## Results

### Baseline characteristics

**Fig. 1:**
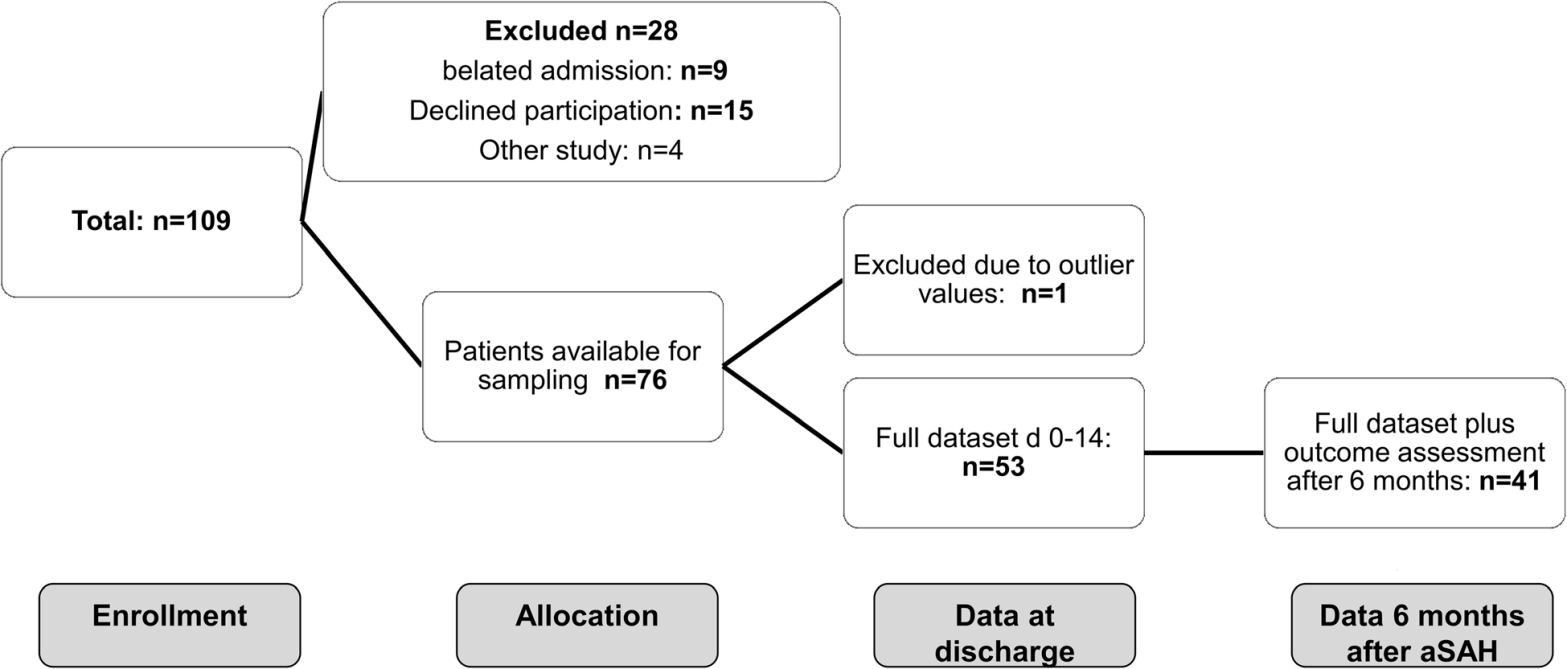
Flowchart of patients enrolled. Diagram of patient enrolment and subsequent data analysis. *aSAH* Aneurysmal subarachnoid hemorrhage

At admission, samples were available for analysis from 76 of the 81 patients (94 %) initially included (Fig. 1). Data for one patient had to be excluded prior to analysis due to an outlier value. Baseline data for the remaining 75 patients are shown in Table 1. Due to patient deaths (total n = 10; 13 % of the patients initially included) or incomplete data (n = 12; 16 % of the patients initially included) the sample size decreased over time leaving data for 53 of 81 patients (65 %) available for analysis on day 14. Complete datasets (measured values for day 0–14) for outcome according to mRS 6 months after aSAH were available for 41 patients (51 %).

**Table 1:** Baseline characteristics

Baseline characteristics	
Number of patients	n = 75
Sex (female)	n = 49 (65 %)
Mean age +/−SD (yrs)	53 +/−13
WFNS:	
WFNS 1	n = 22 (29 %)
WFNS 2	n = 13 (17 %)
WFNS 3	n = 5 (7 %)
WFNS 4	n = 14 (19 %)
WFNS 5	n = 21 (28 %)
Aneurysm treatment:	
Endovascular treatment	n = 52 (69 %)
Surgical treatment	n = 23 (31 %)

*WFNS* World Federation of Neurosurgical Societies score

### Time Course of serum DHEAS levels

**Fig. 2:**
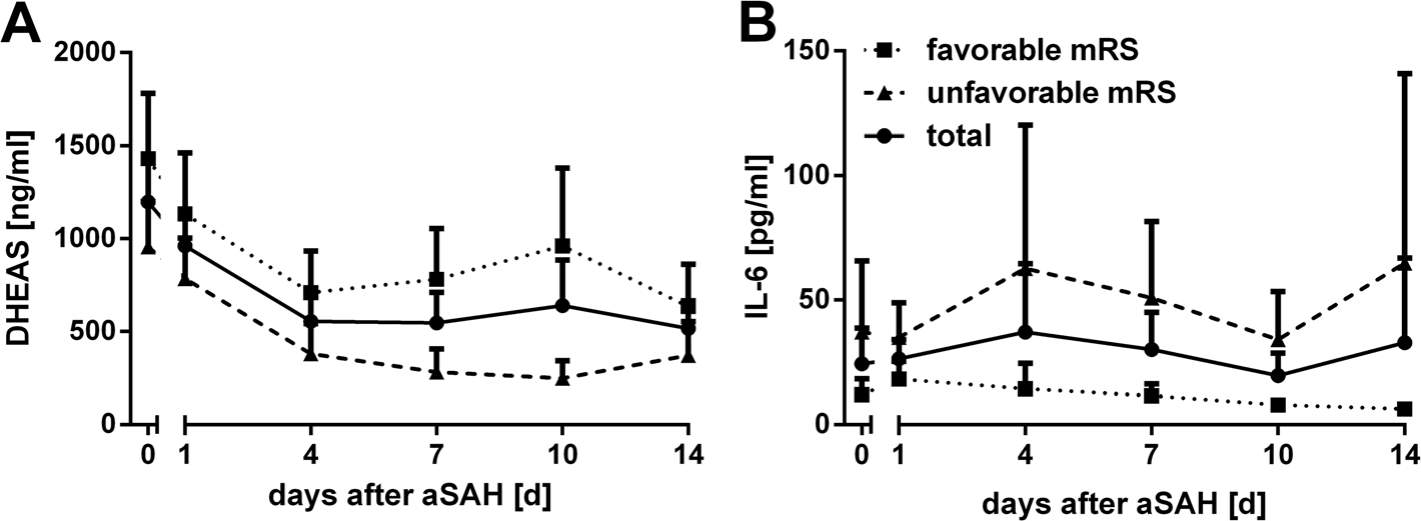
Time course of dehydroepiandrosterone-sulfate (*DHEAS*) and *IL-6* related to outcome at discharge. Time course of *DHEAS* (**a**) and IL-6 (**b**) serum levels (mean + 95 % CI) related to dichotomized outcome at discharge (according to the modified Rankin scale score (*mRS*)

DHEAS serum levels were assessed from day 0 to day 14 of the inpatient stay (always in the morning) (Table 2; Fig. 2).

**Table 2:** DHEAS levels during the inpatient stay

	Minimum	Maximum	Mean	SD	95 % CI	Number
DHEAS day 0	<150	5260	1197	941	884, 1367	75
DHEAS day 1	<150	4460	961	850	671, 1105	73
DHEAS day 4	<150	2840	554	593	363, 675	68
DHEAS day 7	<150	3780	546	670	356, 676	66
DHEAS day 10	<150	5770	639	968	370, 792	62
DHEAS day 14	<150	2560	515	543	360, 666	53

Time course of dehydroepiandrosterone-sulfate (*DHEAS*) levels (ng/ml) during the inpatient stay (up to day 14 after aneurysmal subarachnoid hemorrhage. *Number* number of patients analyzed

As DHEAS levels are dependent on gender and age, additional analysis of data for each subgroup was performed: DHEAS levels over time are shown in relation to gender (Additional file 1: Figure S1A) and to age (<55 years vs >55 years) (Additional file 1: Figure S1B). Mixed ANOVA with gender as a between-subjects effect did not show a significant difference in DHEAS levels (expressed as sqrtDHEAS) (*F* (1, 50) = .155; *p* = .695). However, with age as the between-subjects effect there was a significant difference between the time course of DHEAS levels (expressed as its sqrt) (*F* (1, 50) = 4.5; *p* = .039).

The clinical time course after aSAH is influenced considerably by the severity of injury. Therefore, the time course of DHEAS levels were analyzed related to severity of hemorrhage. As a surrogate parameter classification according to the WFNS grading scale was used (WFNS 1–3 = mild; WFNS 4, 5 = severe). The time course of DHEAS levels did not differ in relation to the severity of injury (Additional file 1: Figure S1C) (*F* (1, 50) = 2.79; *p* = .101).

### DHEAS levels related to outcome at discharge and 6 months after aSAH

To evaluate the possibility of correlation of DHEAS levels on outcome, DHEAS serum levels were analyzed related to patients’ outcome at discharge (expressed via dichotomized mRS) (Table 3, Fig. 2a). Overall, higher serum DHEAS levels were found in the subgroup with favorable outcome at discharge.

**Table 3:** DHEAS levels during the inpatient stay according to functional outcome at discharge (dichotomized modified Rankin Scale score)

	Minimum	Maximum	Mean	SD	95 % CI	Number
DHEAS day 0						
Favorable	<150	5260	1431	1077	1081, 1782	38
Unfavorable	<150	2370	955	730	712, 1199	37
DHEAS day 1						
Favorable	<150	4460	1132	989	802, 1462	37
Unfavorable	<150	2740	785	646	566, 1004	36
DHEAS day 4						
Favorable	<150	2840	710	662	486, 934	36
Unfavorable	<150	1700	223	453	215, 542	32
DHEAS day 7						
Favorable	<150	3780	781	796	507, 1054	35
Unfavorable	<150	1110	282	343	156, 407	31
DHEAS day 10						
Favorable	<150	5770	961	1202	542, 1380	34
Unfavorable	<150	937	247	245	152, 342	28
DHEAS day 14						
Favorable	<150	2560	634	602	405, 863	29
Unfavorable	<150	1520	371	430	189, 553	24

Dehydroepiandrosterone-sulfate (*DHEAS*) levels (ng/ml) during the acute stage of disease according to dichotomized (*Favorable* vs *Unfavorable* modified Rankin Scale score) outcome at discharge. *Number* number of cases analyzed

To additionally assess long-term effects, the correlation between early DHEAS levels and levels at 6-month follow up was analyzed. DHEAS serum levels at days 0–14 were also found to be related to outcome 6 months after aSAH (expressed as the dichotomized mRS; Table 4; Fig. 2b). However, a high drop-out rate hampers the interpretation of the long-term results.

**Table 4:** DHEAS levels during the inpatient stay according to functional outcome 6 months after aneurysmal subarachnoid hemorrhage (dichotomized modified Rankin scale score)

	Minimum	Maximum	Mean	SD	95 % CI	Number
DHEAS day 0						
Favorable	<150	5260	1411	1161	1000, 1823	33
Unfavorable	<150	2310	1052	678	766, 1338	24
DHEAS day 1						
Favorable	<150	4460	1135	1035	768, 1502	33
Unfavorable	<150	2740	820	609	563, 1077	24
DHEAS day 4						
Favorable	<150	2840	699	700	451, 948	33
Unfavorable	<150	1700	515	497	283, 748	20
DHEAS day 7						
Favorable	<150	3780	770	838	467, 1072	32
Unfavorable	<150	1110	339	324	187, 491	20
DHEAS day 10						
Favorable	155	5770	982	1251	523, 1441	31
Unfavorable	<150	776	255	249	127, 383	17
DHEAS day 14						
Favorable	<150	2560	669	604	435, 903	28
Unfavorable	<150	1090	359	342	153, 566	13

Dehydroepiandrosterone-sulfate (*DHEAS*) levels (ng/ml) during acute stage of disease according to dichotomized (*Favorable* vs *Unfavorable*) outcome 6 months after aneurysmal subarachnoid hemorrhage. *Number* number of cases analyzed

To objectify this observation, mixed between-within subjects ANOVA was conducted to compare the time course of DHEAS levels with respect to outcome (expressed as dichotomized mRS). Due to skewed data and to fulfil the demands of sphericity and homogeneity of variances, DHEAS levels were expressed as its sqrt. The between-subjects effect (outcome at discharge) was strongly significant (*F* (1, 50) = 12.98; *p* <.0005) with higher sqrtDHEAS levels over the observation period in patients with favorable outcome at discharge. Although across time the serum DHEAS levels changed significantly (*F* (4.36, 218.19) = 21.81; *p* <.0005), this pattern of change did not differ between the two outcome groups (interaction of DHEAS across time with outcome, *F* (4.36, 218.19) = 1.29; *p* = .272). We demonstrated a different time course of DHEAS levels over time for patients with a favorable outcome at discharge compared to those with an unfavorable outcome (*p* <.0005). As DHEAS levels differed significantly with age, we performed subgroup analysis by patient age (<55 years vs >55 years) (Additional file 2: Figure S2): the time course of DHEAS levels (sqrtDHEAS) in relation to outcome at discharge was significantly different in both groups, but this difference was more pronounced in the younger patients (<55 years, *F* (1, 27) = 8.16; *p* = .008; >55 years, *F* (1, 20) = 4.63; *p* = .044). However, the sample size of the subgroups was very small.

Mixed ANOVA was also conducted for dichotomized outcome assessed 6 months after aSAH. Again, on analysis of the between-subjects effect (outcome 6 months after aSAH) there was a significant difference in sqrtDHEAS levels (*F* (1, 38) = 5.89; *p* = .020) between the two outcome groups. As expected from the analysis of outcome at discharge, longitudinal change in serum DHEAS levels did not differ between the two outcome groups (*F* (5, 190) = 1.39; *p* = .229), but sqrtDHEAS serum levels changed significantly over time (*F* (5, 190) = 13.7; *p* <.0005). Subgroup analysis of the outcome 6 months after aSAH was not performed due to the small sample size.

These results suggest a distinct time course of DHEAS levels in patients with a favorable outcome compared to those with an unfavorable outcome. Specifically, there is evidence for higher DHEAS serum levels across time in patients with a favorable outcome at least when assessed at discharge.

### Time course of serum IL-6 levels

**Fig. 3:**
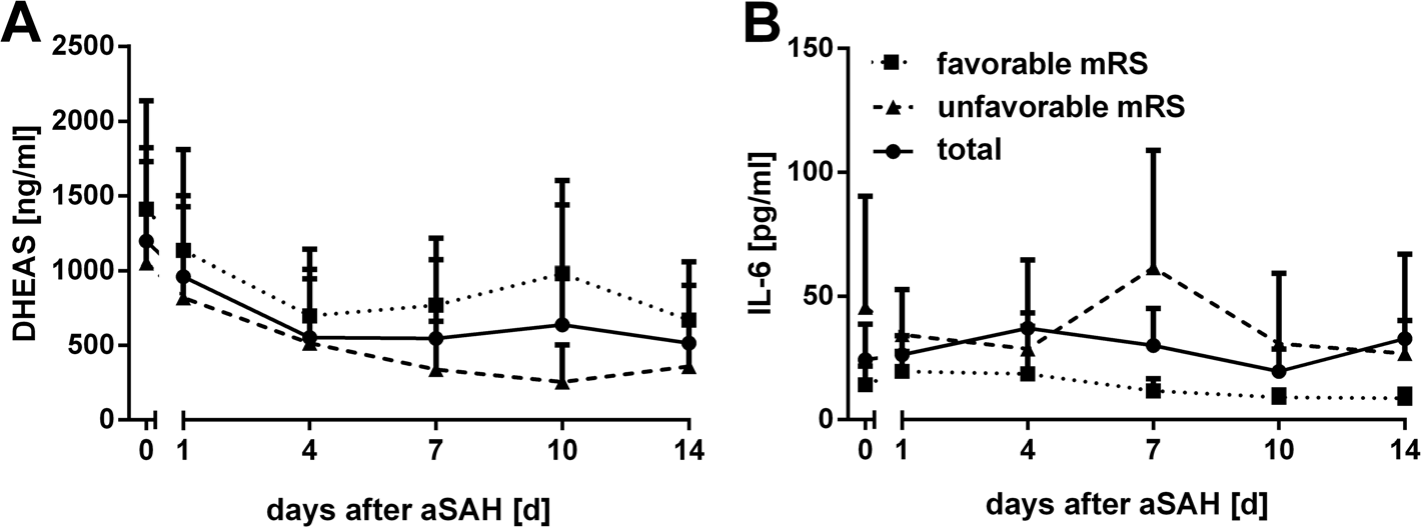
Time course of dehydroepiandrosterone-sulfate (*DHEAS*) and IL-6 related to outcome 6 months after aneurysmal subarachnoid hemorrhage (*aSAH*). Time course of DHEAS (**a**) and IL-6 (**b**) serum levels (mean + 95 % CI) related to dichotomized outcome 6 months after aSAH (according to the modified Rankin scale score (*mRS*))

Serum IL-6 levels were also measured over the whole observation period (days 0–14, always in the morning) (Table 5, Fig. 3).

**Table 5:** IL-6 levels during the inpatient stay

	Minimum	Maximum	Mean	SD	95 % CI	Number
IL-6 day 0	<2	529	24	63	10, 39	75
IL-6 day 1	<2	194	26	33	19, 34	73
IL-6 day 4	<2	878	37	113	10, 65	68
IL-6 day 7	<2	413	30	61	15, 45	66
IL-6 day 10	<2	241	20	36	11, 29	62
IL-6 day 14	<2	905	33	124	−1, 67	53

Time course of IL-6 levels (pg/ml) during the inpatient stay (up to day 14 after aneurysmal subarachnoid hemorrhage). *Number* number of cases analyzed

Analogous to DHEAS, serum IL-6 levels across time (Table 5, Fig. 3) were also related to outcome according to dichotomized mRS at discharge and 6 months after aSAH (Tables 6 and 7, Fig. 3b).

**Table 6:** IL-6 levels during the inpatient stay according to functional outcome at discharge (dichotomized modified Rankin scale score)

	Minimum	Maximum	Mean	SD	95 % CI	Number
IL-6 day 0						
Favorable	<2	109	12	20	6, 18	38
Unfavorable	<2	529	37	86	9, 66	37
IL-6 day 1						
Favorable	<2	122	18	22	11, 25	37
Unfavorable	3	194	35	40	21, 49	35
IL-6 day 4						
Favorable	<2	175	14	30	4, 25	36
Unfavorable	<2	878	63	160	5, 120	32
IL-6 day 7						
Favorable	<2	53	12	14	7, 16	35
Unfavorable	<2	413	51	84	20, 82	31
IL-6 day 10						
Favorable	<2	33	8	8	5, 11	35
Unfavorable	3	241	34	50	15, 53	28
IL-6 day 14						
Favorable	<2	30	6	6	4, 9	29
Unfavorable	2	905	65	181	−12, 141	24

IL-6 levels (pg/ml) during the acute stage of disease according to dichotomized (*Favorable* vs *Unfavorable*) outcome at discharge. *Number* number of cases analyzed

**Table 7:** IL-6 levels during the inpatient stay according to functional outcome 6 months after aneurysmal subarachnoid hemorrhage (dichotomized modified Rankin scale score)

	Minimum	Maximum	Mean	SD	95 % CI	Number
IL-6 day 0						
Favorable	2	109	14	21	7, −22	33
Unfavorable	<2	529	46	16	1, 90	24
IL-6 day 1						
Favorable	<2	122	20	23	11, 28	32
Unfavorable	3	194	34	43	16, 53	24
IL-6 day 4						
Favorable	<2	315	19	55	−1, 38	33
Unfavorable	5	129	29	31	14, 43	20
IL-6 day 7						
Favorable	<2	60	12	13	7, 17	32
Unfavorable	<2	413	61	101	14, 109	20
IL-6 day 10						
Favorable	<2	33	9	9	6, 12	32
Unfavorable	<2	241	31	56	2, 59	17
IL-6 day 14						
Favorable	<2	42	9	10	5, 12	28
Unfavorable	6	75	27	22	13, 40	13

IL-6 levels (pg/ml) during the acute stage of disease according to dichotomized (*Favorable* vs *Unfavorable*) outcome 6 months after aneurysmal subarachnoid hemorrhage. *Number* number of cases analyzed

As the severity of injury also might influence IL-6 secretion, the time course of serum IL-6 levels was analyzed in relation to the dichotomized WFNS score. Especially during the early time course, serum IL-6 levels differed visibly (Additional file 3: Figure S3). Objectified by mixed ANOVA, serum IL-6 levels (expressed as logIL-6) varied over time in relation to the WFNS score (*F* (1, 43) = 8.62; *p* = .005).

Corresponding to the DHEAS analysis, mixed between-within subjects ANOVA was conducted to evaluate IL-6 serum levels across time in relation to outcome. Due to skewed data, serum logIL-6 levels were calculated prior to analysis. Across time, the logIL-6 levels did not change significantly (*F* (3.97, 170.84) = 1.94; *p* = .116). Comparing the two outcome groups at discharge (favorable vs unfavorable) the pattern of logIL-6 levels did not differ between the groups (*F* (3.97, 170.84) = .886; *p* = .473). Of note, the between-within subject effect indicating different time courses of logIl-6 levels in relation to the dichotomized outcome score (favorable vs unfavorable) was strongly significant (*F* (1, 43) = 32.08; *p* < .0005). Thus, these results suggest a difference in IL-6 levels, with higher IL-6 levels across time in the group with an unfavorable outcome.

In the subgroups with WFNS 1–3 vs WFNS 4, 5, there was a significant difference in the time course of serum IL-6 (logIL-6) levels in relation to outcome at discharge in both subgroups (mild hemorrhage WFNS 1–3, *F* (1, 20) = 5.08; *p* = .036; severe hemorrhage WFNS 4, 5, F (1, 21) = 16.85; *p* = .001), but again the sample size was small (Additional file 4: Figure S4).

For outcome assessed 6 months after aSAH, no significant main effects were noted for time (*F* (4.68, 154.4) = .948; *p* = .448) or interaction of time and outcome after 6 months (*F* (4.68, 154.4) = .878; *p* = .492). Again, the main effect comparing the two outcome groups was significant (*F* (1, 33) = 17.15; *p* <.0005) suggesting a difference between the outcome groups in IL-6 levels over time, with higher IL-6 levels in the group with an unfavorable long-term outcome.

## Discussion

A favorable outcome at discharge and 6 months after aSAH was related to higher DHEAS serum levels during the acute treatment period after aSAH. Inversely, serum levels of IL-6 were considerably lower in patients with a favorable outcome. Thus, we might assume that there is a functional link between the anti-inflammatory properties of DHEAS and the less pronounced inflammatory reaction in terms of IL-6 expression after aSAH, resulting in an improved outcome.

DHEAS has demonstrated multiple neuroprotective effects [14–16, 27]. Multiple mechanisms of action have been suggested, such as modulation of Gamma-aminobutyric-acid-A(GABA_A_)-receptor signaling [15, 28], inhibition of N-methyl-D-aspartate (NMDA)-receptor function, sigma receptor or glutamate receptor signaling [12, 29]. Genomic and non-genomic signaling via direct interaction with postsynaptic receptors has been demonstrated [30]. However, the mechanism of action remains uncertain and seems to be versatile, resulting in multiple effects, such as anti-inflammatory action. In fact, sex hormones modulate the immune system [31, 32]. An influence on IL-6 synthesis has been demonstrated specifically for DHEAS [33, 34]. Apart from physiological derangements (such as elevation of intracranial pressure and reduction of cerebral blood flow), oxidative and metabolic disturbances, pathological acute vascular reactions, ionic changes etc., inflammatory reactions arise early after aSAH [35, 36]. Currently, it is assumed that these pathological mechanisms contribute largely to the development of secondary injuries and, finally, to the limited chance for favorable outcome after aSAH. Lower IL-6 levels were shown to be related to an improved outcome [25]. Elevated DHEAS and reduced IL-6 serum levels measured in patients with a favorable outcome possibly indicate a protective hormonal and inflammatory pattern. This effect may be assumed to be the result of the anti-inflammatory properties of DHEAS leading to decreased IL-6 synthesis, thus confirming the well-known link between the neuroendocrine and immune network [31].

Additionally, decreased DHEA(S) levels have been related to the risk of cardiovascular diseases, reducing the incidence of cardiovascular pathologies [13, 37]. Vasoprotective properties inhibiting remodeling after vascular injury have been observed in experimental studies [38]. However, the results of published clinical studies are conflicting [39]. Despite these uncertainties, neurosteroids are still regarded as promising therapeutic agents because there is substantial experimental evidence for beneficial cardiovascular effects due to anti-inflammatory, vasorelaxant and anti-remodeling properties [40, 41]. In a model for vascular remodeling, DHEA alleviates oxidative stress and inflammation with decreased IL-6 mRNA expression via inhibition of p38 mitogen-activated protein kinase/nuclear factor κB (p38 MAPK/NF-κB) [38]. These vasoprotective properties, which ameliorate oxidative and inflammatory stress after vascular lesions, may also contribute to the suspected beneficial effect of higher DHEAS serum levels after aSAH. Thus, an anti-inflammatory reaction by DHEAS resulting in IL-6 synthesis could also occur locally, i.e. directly at the affected vessels.

Limitations of this study arise from its relatively small and inhomogeneous cohort (both sexes, wide age range, aSAH graded WFNS 1–5). Therefore, standard variations are considerably high. Furthermore, there was a substantial drop-out rate in relation to the outcome assessment 6 months after aSAH. Patients lost to follow up are most likely those with an unfavorable outcome. Therefore, our long-term results are biased by the response rate 6 months after aSAH and are insufficient to assess the relationship between long-term outcome and the time course of DHEAS levels. More reliable results may be obtained by a follow-up study with a larger and more homogeneous cohort as well as a tighter follow-up schedule.

Only DHEAS but not DHEA or androstenedione (ASD) was assessed. DHEAS forms the pool for DHEA and ASD. Furthermore, conversion of downstream steroid hormones is based on the presence of DHEAS. Therefore, DHEAS levels reflect the reservoir for further synthesis. Another advantage of choosing DHEAS as a marker for the presence of neuroactive steroid is its stability and feasibility of detection. Thus, as already shown by various previous studies, we favor the approach of determining DHEAS as a surrogate marker for neuroactive steroids.

Outcome assessed according to the mRS only roughly reflects the neurological and functional status of the patients. Moreover, outcome scores were dichotomized for analysis. Thus, subtle discrete changes in outcome are not detectable by this approach. Age and gender influence DHEAS levels: subgroup analyses were performed to rule out major flaws and even if the age-dependent subgroups are examined, DHEAS levels over time differed in relation to outcome at discharge. However, the sample size for subgroup analysis was too small to make a firm conclusion.

The same applies to IL-6 serum levels: an increase in IL-6, especially when measured in serum, may reflect an unspecific response to stress and cytokine production, may be related more to a global inflammatory reaction than to a specific pathophysiological mechanism involved in early brain injury after aSAH. Nevertheless, in line with our initial hypothesis, subgroup analysis according to WFNS score also revealed a significantly different time course for serum IL-6 levels in patients with an unfavorable outcome compared to those with a favorable outcome. Alhough serum IL-6 probably does not reflect the extent of neuroinflammation after aSAH in detail, it may serve as an easily accessible (though unspecific) surrogate parameter for global inflammatory burden after aSAH. However, regardless of its final interpretation, use of serum IL-6 instead of cerebral (measured by microdialysis) or cerebrospinal fluid IL-6 is indispensable in relating the results to DHEAS levels, which have also been measured in serum.

The time course of DHEAS and IL-6 is difficult to interpret. There is a distinct pattern for IL-6 with a peak at day 4 followed by a slow decrease. By contrast, DHEAS dropped towards day 4 and roughly remained at the current level. Though the time course of IL-6 and that of DHEAS behave conversely, the mechanism behind these patterns remains unidentified. Occurrence of both delayed cerebral ischemia (DCI) and death during the inpatient time course in our patient group peaked after day 4 (DCI day 7, death day 10). Even if regarded as an early biomarker peak, the IL-6 levels at day 4 followed by a decrease are not plausibly explained by the occurrence of DCI at day 7, neither by death at day 10. Finally, mostly descriptive data are presented. Based on the complexity of interactions, skewed data, confounders and the dynamic acquisition of data, the data analysis and interpretation is difficult and error-prone when evaluating a small and inhomogeneous cohort.

## Conclusion

Taken together, we present for the first time the serum profiles of DHEAS and IL-6 during the acute stage after aSAH and their relationship to outcome. There is evidence that DHEAS exerts protective effects with improvement of functional outcome after aSAH, possibly by influencing the inflammatory response after aSAH by decreasing IL-6 serum levels. Further studies with a larger sample size are necessary to better adjust for confounders such as age, gender or smoking status.

## Key messages

- Higher DHEAS levels may be beneficial to functional outcome after aSAH.
- The possible action of mechanism may be a modulation of the inflammatory response after aSAH by DHEAS, resulting in decreased IL-6 serum levels.

## Additional files

Additional file 1: Figure S1. Dehydroepiandrosterone-sulfate (*DHEAS*) levels related to gender (**A**), age *<55 years* vs >*55 years* (**B**) and World Federation of Neurosurgical Societies (*WFNS*) score at admission (*WFNS 1*–*3* vs *WFNS 4*–*5*) (**C**). *aSAH* Aneurysmal subarachnoid hemorrhage. Additional file 2: Figure S2. Dehydroepiandrosterone-sulfate (*DHEAS*) related to gender with respect to outcome at discharge. **A** Patient cohort *<55 years* of age related to outcome. **B** patient cohort *>55 years* of age related to outcome. *aSAH* Aneurysmal subarachnoid hemorrhage, *mRs* Modified Rankin scale. Additional file 3: Figure S3. IL-6 levels related to initial World Federation of Neurosurgical Societies (*WFNS*) score (*WFNS 1*–*3* vs *WFNS 4*–*5*). *aSAH* Aneurysmal subarachnoid hemorrhage. Additional file 4: Figure S4. IL-6 levels related to initial World Federation of Neurosurgical Societies (*WFNS*) score with respect to outcome at discharge: **A** Patients with initial *WFNS* score *1*–*3*. **B** Patients with initial *WFNS* score *4*–*5. DHEAS* Dehydroepiandrosterone-sulfate.

## Abbreviations

ANOVA: Analysis of variance
aSAH: Aneurysmal subarachnoid hemorrhage
ASD: Androstenedione
DHEA: Dehydroepiandrosterone
DHEAS: Dehydroepiandrosterone-sulfate
GABA_A_: Gamma-aminobutyric-acid-A
IL-6: Interleukin 6
LOD: Lower limit of detection
log: Logarithm
mRNA: Messenger ribonucleic acid
mRS: Modified Rankin Scale score
NF-κB: Nuclear factor κB
NMDA: N-methyl-D-aspartate
p38 MAPK: p38 mitogen-activated protein kinase
sqrt: Square root
WFNS: World Federation of Neurosurgical Societies
